# The Physiopathological Link Between Bisphenol A Exposure and Molar Incisor Hypomineralization Occurrence: A Systematic Review

**DOI:** 10.3390/dj13080332

**Published:** 2025-07-22

**Authors:** Estelle Mathonat, Thibault Canceill, Mathieu Marty, Alison Prosper, Alexia Vinel, Emmanuelle Noirrit-Esclassan

**Affiliations:** 1 Odontology Department, Health Faculty, Toulouse University, 3 Chemin des Maraichers, 31400 Toulouse, France; 2 Odontology and Oral Medicine Department, Toulouse Universitary Hospital, 2 rue de Viguerie, 31100 Toulouse, France; 3 Intestine ClinicOralomics Intestine Microbiota and Metabolism (InCOMM) Team, French Institute of Metabolic and Cardiovascular Diseases (i2MC), Inserm UMR1297, 1 Avenue Jean Poulhès, 31400 Toulouse, France; 4 Perinatal, Paediatric and Adolescence Health: Evaluative and Epidemiologic Approach Team (SPHERE), Centre for Epidemiology and Population Health Research (CERPOP), Inserm UMR1295, 37 Allées Jules Guesde, 31000 Toulouse, France; 5 Dental Medicine School of Marseille, 27 Boulevard Jean Moulin, 13385 Marseille, France

**Keywords:** MIH, molar incisor hypomineralization, BPA, Bisphenol A, endocrine disrupting chemicals

## Abstract

**Objective**: This study aimed to assess, through a systematic review, the potential link between bisphenol A (BPA) exposure and molar incisor hypomineralization (MIH). **Methods**: A systematic review was performed according to the PRISMA grid. All international studies—in vitro, in vivo, or clinical—evaluating the relationships between bisphenol A and MIH were included. An iterative search of eligible publications was conducted on May 26, 2025, using three different databases: PubMed, Science Direct, and Google Scholar. **Results**: Eleven studies were included in the review, ten of which were experimental studies. They were published between 2013 and 2024. Among the selected articles, a rat model was used in eight studies and seven established a link between MIH and BPA (63.64% of the articles). In the included studies, the incisors of rats treated with BPA presented asymmetrical white spots at the enamel level, with a phenotype similar to human MIH. The authors highlight the hypothesis of the implication of steroid receptors expressed by ameloblasts, in particular at the stage of maturation, thus impacting enamel quality. **Conclusions**: The results presented in this review highlight a trend in the interaction of bisphenol A with steroid receptors, thus affecting enamel quality. However, these associations are weak, and future studies should investigate cofactors modulating BPA’s role in the development of MIH.

## 1. Introduction

Molar incisor hypomineralization, more commonly known as MIH, is an enamel defect characterized by the hypomineralization of at least one of the first four permanent molars associated or not with the involvement of the permanent incisors [1]. MIH is a daily concern for dental practitioners [2] as it is estimated that more than one in ten children have MIH, according to a 2018 global prevalence study [3].

The enamel lesions are often asymmetrical and white to brown in color, and they sometimes lead to structural losses in severe forms [4]. This qualitative anomaly is acquired during the period of crown mineralization of permanent incisors and molars, extending from the third trimester of fetal life to 3 years after birth. Initially described on permanent teeth, similar lesions have also been found on second primary molars. This impairment is now called HSPM, or “hypomineralized second primary molars”. Several studies have shown that children with HSPM would be more likely to develop MIH later, especially those with severe forms of HSPM [5,6].

Difficult to treat, this disease often causes strong sensitivities in children. For the most affected teeth, extraction can be considered. Faced with these observations, asking the question of etiology becomes essential. Though many suggestions have already emerged, there is no consensus today: MIH is thus qualified as a multifactorial pathology. It is linked to a combination of systemic, medical, genetic, and environmental factors. The literature identifies multiple potential etiologies such as maternal illness, perinatal hypoxia, childhood illnesses (such as respiratory infections, chickenpox) associated with hyperpyrexia, vitamin D/A deficiencies, and dioxin/BPA exposure [7].

**Figure 1:**
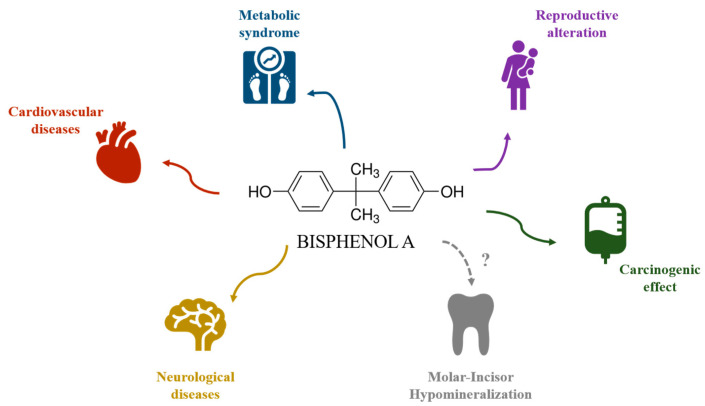
Harmful implications of bisphenol A for human health.

However, as the mineralization of permanent teeth takes place during a very specific period of time, considerations of the topic may be concentrated on a potential causative agent acting during this specific period. Thus, environmental factors, and particularly endocrine disruptors such as bisphenol A (BPA), may play an important role in MIH occurrence. BPA is an environmental xenoestrogen found in many consumer products, leading to widespread exposure. Research has shown that BPA can interfere with various biological systems, contributing to a range of health issues (Figure 1). The compound’s ability to mimic hormones and bind to estrogen receptors is central to its disruptive effects [8]. It is now recognized for its adverse health effects on female reproductive function [9], mammary gland development [10], cognitive function, and metabolism. Through its effects on body weight regulation, BPA can contribute to cardiovascular diseases [10,11], and the compound’s interaction with various receptors and transcription factors influences fat and liver homeostasis, exacerbating metabolic syndromes [10]. BPA exposure has also been shown to impair neurological functions, leading to structural and molecular changes in the brain. It is linked to neurodevelopmental disorders like autism and neurodegenerative diseases, as it promotes oxidative stress and neuroinflammation and disrupts neurotransmitter functions, contributing to cognitive and emotional disorders [12]. In 2017, BPA was classified as a substance of very high concern (SVHC) by the European Chemicals Agency and its regulation system REACH (for Registration, Evaluation, Authorization and Restriction of Chemicals) because it exhibits carcinogenic properties by influencing receptor activity and inducing oxidative stress, which can lead to DNA damage and cancer progression [13].

BPA is widely used in industry for the manufacture of polycarbonate and epoxy resins, and it is also found in a derived form in some dental materials (composite resins…) [14]. The daily exposure of an individual is therefore particularly important, especially through the consumption of canned meat, vegetables, and drinks [15]. This concerns children as well as adults and especially pregnant women. Sensitivity to BPA is highly elevated during the perinatal period because BPA is able to cross the placental barrier and affect the fetus [16,17].

The objective of this work is to assess, through a systematic review of the literature, a potential link that may exist between exposure to bisphenol A and the development of MIH. The problem is to attempt to understand how BPA may affect enamel mineralization.

## 2. Materials and Methods

The methodology chosen to conduct this study is a systematic review of the literature. It has been designed, as reported here, according to the PRISMA grid [18,19].

### 2.1. Eligibility Criteria

All articles that have reported in vitro, in vivo, or clinical evaluation of the relationships between bisphenol A and MIH were included. Publications dealing with hypomineralization other than enamel delineated opacities were not included. Studied could include male, female, or mixed-sex animals or humans. Following the first stage of inclusion, articles that were not in English were planned to be excluded, but this did not occur.

### 2.2. Information Sources

The iterative search of eligible publications was conducted on 26 May 2025 on three different databases according to recommendations [20]: PubMed, Science Direct, and Google Scholar. No time limit was set in the request.

### 2.3. Search Strategy

The query published in PubMed is presented in Appendix A.1. As the Science Direct and Google Scholar websites do not allow the application of the same request as designed for PubMed, the procedure submitted was as follows: ((MIH) OR (molar incisor hypomineralization) OR (molar-incisor hypomineralization)) AND ((endocrine disruptor) OR (BPA) OR (BPS) OR (Bisphenol)). The acronym BPS was used, as bisphenol S is often studied in parallel with BPA in publications.

### 2.4. Selection Process

The titles and abstracts of the publications obtained in the results of the requests on the two databases were reviewed by two evaluators to determine whether or not they met the eligibility criteria mentioned above. When in doubt, the full text was analyzed to determine if the article was suitable for inclusion.

### 2.5. Data Collection Process and Items

The analysis of the full texts of the included publications made it possible to identify and group the following information in an Excel spreadsheet (Microsoft Excel 2010^®^): first author’s name and geographical origin, year of publication, journal and impact factor, type of study and duration, main objective, primary outcome, experimental protocol, number of samples, animals or individuals included, and main outcomes obtained.

### 2.6. Summary of Results

Because of the high diversity of outcomes in the included publications, this review cannot draw up a meta-analysis of the results already published in the literature. A summary table of the data concerning the possible relationship between exposure to BPA and the occurrence of MIH will be proposed. The presentation of results in paragraphs will be based on the recommendations of the PRISMA grid, the complete form of which is available in Appendix A.2.

### 2.7. Bias Assessment

The risk of bias has been assessed by one evaluator with official surveys created by Joanna Briggs Institute (JBI) for clinical cross-sectional studies [21] and quasi-experimental studies [22], which have already been described for bias assessment in systematic reviews of in vitro studies [23].

## 3. Results

### 3.1. Study Selection

**Figure 2:**
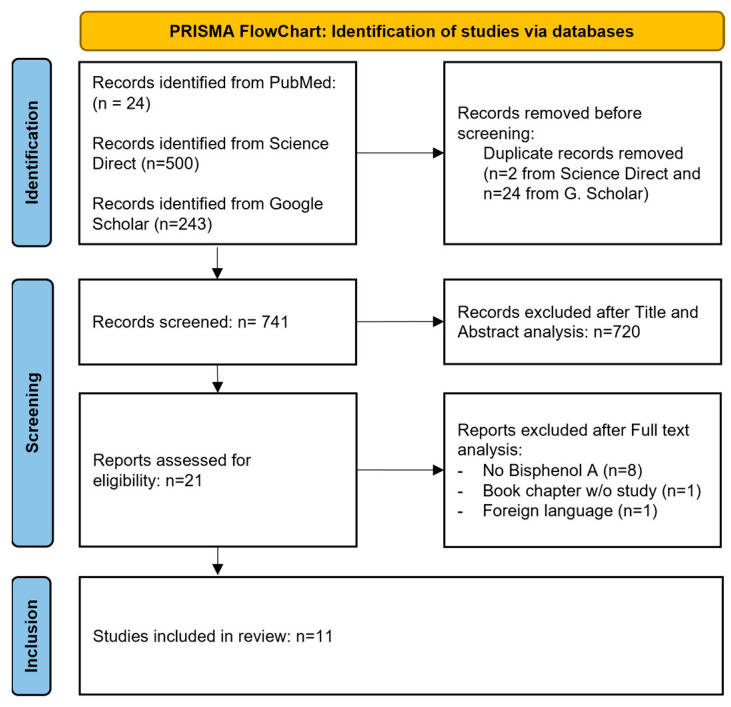
PRISMA flowchart of the review.

The PubMed search returned 24 articles, 9 of which met the inclusion criteria. Regarding the Science Direct database, 500 articles were obtained but only 2 met the inclusion criteria. These articles were also retrieved from PubMed, so no additional publications were added after the querying of Science Direct. The Google Scholar database proposed 243 research results, among which 2 have been retained for inclusion (Figure 2).

### 3.2. Main Characteristics of the Selected Studies

Ten of the eleven articles included in this review describe experimental studies, and one is a clinical retrospective study. Eight of them (72.73%) were carried out by the same team of researchers from the Laboratory of Molecular Oral Pathophysiology (Inserm UMR1138, Paris, France). The selected articles were published from 2013 to 2024 and reported no conflicts of interest (we do not have information for the conference abstract of Ai Thu et al. [24]). The average impact factor of the journals in which the studies were published was 3.95 ± 1.45 (*n* = 9, with one of the articles being published in a journal without an impact factor and the other being a conference abstract, which has not been taken into account).

Among the selected articles, eight use a rodent model (the rat) (72.73% of the studies), one a mouse model (9.09%), and the other a mussel model (9.09%). The number of rats included varies from 3 to 16 in each group depending on the study, with an average of 9.25 ± 5.09 animals per group when combining studies. Seven of the articles established a link between incisor enamel hypomineralization, with a pattern similar to that of MIH, and BPA (63.64% of the articles). The clinical retrospective study, published in 2024, described a positive, but not significant, correlation between the two parameters.

### 3.3. Results of Individual Studies

The analysis of the included articles reveals that bisphenol A (BPA) exposure is weakly but positively correlated with dental hypomineralization, though not statistically significant. BPA is toxic to zebra mussels, disrupting shell biomineralization. BPA and sodium fluoride reduce protease expression, inhibit crystal growth, and cause enamel hypomineralization. The androgen signaling pathway is critical throughout enamel mineralization, with peak androgen receptor expression in maturation-stage ameloblasts. BPA and vinclozolin exert anti-androgenic effects, primarily in males, impairing enamel maturation. Steroid receptor expression in ameloblasts during maturation influences enamel quality. Moreover, male rats exhibit greater alterations in kallikrein 4 and enamelin expression than females. The nine articles included in the review are detailed further in Table 1.

### 3.4. Bias Assessment

The bias evaluation of the included articles shows their excellent quality (Table 2). Only the clinical study, which is retrospective on a large database, has a quality score below 70%, although its risk of bias remains moderate. Except for the conference abstract proposed by Ai Thu et al. [24], all in vivo and in vitro studies are at low risk of bias with scores ≥ 85%. As the selected studies focused on the occurrence of pathology according to exposure to an endocrine disruptor, the authors systematically differentiated male and female animals. This choice is relevant in view of the differences in hormonal and endocrine systems according to sex, and avoids generating errors in the interpretation of results.

## 4. Discussion

The studies included in this review suggest that BPA—and potentially other endocrine disruptors—may contribute to MIH, although associations are weak. The discovery of the expression of many steroid receptors by ameloblasts has shown that BPA can affect enamel quality in rodents, in particular through these receptors, by mimicking natural hormones such as estrogens or androgens. Many factors can thereby disrupt these signaling pathways and impact amelogenesis.

The teeth of rats exposed to BPA have structural and biochemical characteristics similar to MIH observed in humans. Throughout BPA exposure, genes expressed during amelogenesis led to an increase in the synthesis of enamelin by ameloblasts, with the level of expression being decisive for the structure and quality of future enamel [34]. A decrease in the synthesis of kallikrein-related peptidase 4 (KLK4), a protease involved in the breakdown of enamel proteins, was also observed [34]. These two protein variations, essential for proper development, could therefore lead to hypomineralization.

While the studies included in this review showed that BPA could be a potential causative factor of enamel hypomineralization in humans, it seemed essential to study the association with other endocrine disruptors. People are constantly confronted with this “cocktail effect” of EDs, making any forecast difficult, if not impossible, as the number of factors at play is important. Thus, studies have shown that low doses of BPA combined with low doses of Genistein (phytoestrogen) and Vinclozolin (fungicide with anti-androgenic effect) did not lead to an increase in hypomineralization [33]. Moreover, the combination of fluoride and BPA [28], or also of phthalates plus BPA, exacerbated enamel defects [24]. Interestingly, it has also been noted that BPA’s effect on enamel preferentially impacts male rats [29]. This sexual dimorphism is not always shown in human epidemiological studies and mainly depends on the type of endocrine disruptor considered. For example, in a study published in 2023, Boyer et al. found a greater risk for boys to develop MIH when they are exposed to intermediate levels of perfluorooctanoic acid (PFOA) and perfluorooctane sulfonate (PFOS) [35]. The only clinical study included in our review attempting to establish a link between ED exposure and MIH showed a positive but non-significant correlation between the two [25]. The authors’ work on a database limited the extent to which confounding factors could be taken into account and, therefore, the precision of the results. With a prospective study not being ethically feasible when it comes to exposing pregnant women or young children to products containing endocrine disruptors, retrospective studies are still the most promising, with their known biases. What is more, clinical studies on the subject often struggle to conclude whether or not there is a direct causal link between a chemical substance and an enamel defect, as the exposure times and the multitude of cofactors involved vary from one individual to another [36].

Hypomineralization occurs due to the disruption of a small number of genes, a disruption that can be extended to many endocrine disruptors other than BPA [37]. According to a study published in 2017 by Babajko et al., the involvement of the steroid axis in amelogenesis could help explain the etiologies now put forward for MIH [38]. Almost all the etiological hypotheses of MIH (such as vitamin D deficiency or prolonged breastfeeding…) would therefore be directly or indirectly linked to endocrine disruptors.

Given this observation of a potential link between exposure to BPA and the occurrence of enamel hypomineralization, but also all the other repercussions on humans recently demonstrated (decreased fertility, hormone-dependent cancer, behavioral disorder, diabetes, obesity...), the European Chemicals Agency (ECHA) classified bisphenol A, bisphenol B, and 2,2-bis(4′-hydroxyphenyl)-4-methylpentane as “substances of very high concern” in April 2022. The objective of this classification is to reduce their use and replace them with safer products. BPA is unfortunately substituted today by several substances of the bisphenol family (BP-B, S, F), which also appear to be dangerous [39,40,41]. These bisphenols are almost unregulated today: in Europe, BPS is authorized in plastics and articles in contact with food, although this is not the case with BPF. Regulations are still lagging behind the pace of the commercialization of chemicals and their diversity. Thus, among thousands of chemical substances likely to be endocrine disruptors, only a hundred are covered by the European REACH regulation.

For the first time, the Esteban 2014–2016 national program made it possible to measure, in the French population, the levels of impregnation of BPA, S, and F. They were detected in almost all the urine samples tested. Moreover, bisphenol impregnation was higher in children than in adults [42]. The substitution of BPA is therefore a concern. Even if its prohibition appeared effective in 2011, the substitutes used do not seem to be devoid of harmful effects on humans. It may therefore be possible to assume an interaction similar to BPA and thus, potentially, enamel damage such as MIH.

It should be noted that BPA is also present in dentistry. Even if the majority of BPA contamination occurs through food, the potential oral contamination linked in particular to dental resins is also to be taken into account. BPA is not found in its pure state but in a derivative form in composites, i.e., Bis-GMA, Bis-EMA, or Bis-DMA monomers [43]. As a precautionary measure and pending conclusive results, Fleisch et al. recommended limiting the use of composite resins during pregnancy [44] in 2010.

## 5. Conclusions

Through the results presented in this review, we have shown a weak interaction of bisphenol A with steroid receptors, as BPA binds to estrogen receptors in ameloblasts and interferes with androgen receptor functioning. Therefore, BPA may specifically affect amelogenesis depending on the activated receptors, giving rise to different phenotypes and a variable profile of MIH according to the individual. However, the association between BPA exposure and MIH remains weak, and many cofactors should now be studied in the development of this enamel disease. On the other hand, the potential for MIH to be considered an early marker of exposure to endocrine disruptors such as BPA should be studied. It seems relevant to train dentists in the early detection of MIH in children, not only in the logic of care, but especially in the prevention of the long-term deleterious effects of endocrine disruptor exposure.

## Figures and Tables

**Table 1:** Summary of information identified in the articles included in this study.

Year	Authors	Country	Objective	Study Type	Male (Yes/No)	Female (Yes/No)	Number of Subjects	Cellular Analyses (Yes/No)	Conclusion
2024	Winkler et al. [25]	USA	To investigate the relationship between intrauterine exposure to ED and enamel developmental defects in children.	Clinical retrospective study	Yes (53%)	Yes (47%)	356	No	Weak positive correlations (but not statistically significant) were found between an increasing concentration of BPA and an increased number or proportion of teeth with hypomineralization.
2022	Liu et al. [26]	Germany	To investigate the feasibility of zebra mussel (*Dreissena polymorpha*) as a novel model to screen potential MIH-related factors.	In vivo			46 groups of 7		BPA was toxic to zebra mussels and interfered quantitively with shell biomineralization. Nacre is composed of 95% mineral (aragonite, not hydroxyapatite) with an organic matrix, similarly to tooth enamel.
2022	Duman et al. [27]	Turkey	To investigate how prenatal environmental factors (e.g., BPA) affect AMELX and AMBN production of ameloblasts.	In vivo	No	Yes (100%)	15 (5 groups of 3)	No	Abnormal enamel matrix formation was observed in all experimental groups, including those with BPA exposure. AMELX and AMBN staining was significantly lower than that of the control.
2017	Ai Thu et al. [24]	France	To compare mouse enamel defects resulting from exposure to low-dose BPA and/or phthalates; To elucidate the mechanism of action of both endocrine disruptors during amelogenesis.	In vivo (conference abstract)	Unknown	Unknown	Unknown	Yes	Depending on their nature and the time of exposure, phthalates and BPA could affect enamel quality and/or quantity.
2016	Jedeon et al. [28]	France	To study the impact of exposure to BPA and sodium fluoride (NaF) on fluorosis and hypomineralization.	In vivo and in vitro	Yes	Yes	4 groups of 12 rats	Yes	BPA and NaF decrease the expression of proteases and disrupt pH, causing the inhibition of crystal growth and thus the hypomineralization of enamel.
2016	Jedeon et al. [29]	France	To explore androgen receptors due to the preferential enamel impact of BPA on male rats.	In vivo and in vitro	Yes	No	8 groups of 8 rats	Yes	The androgen signaling pathway is involved throughout the enamel mineralization process. The highest expression of androgen receptors is in maturation-stage ameloblasts. BPA and V exert an anti-androgenic effect, preferentially in male rats, which can specifically affect enamel.
2016	Houari et al. [30]	France	To explore the molecular pathways stimulated by BPA during amelogenesis and the different receptors known to regulate the effects of BPA.	In vivo and in vitro	Yes	Yes	6 groups of 3 rats.	Yes	Many steroid receptors are expressed by ameloblasts, in particular at the stage of maturation, impacting enamel quality rather than quantity. A parallel can be made with MIH pathology, especially involving enamel quality, and, therefore, the final stages of enamel synthesis.
2016	Jedeon et al. [31]	France	To assess differences in hypomineralization between female and male rats following exposure to 3 EDs (V, G, and BPA).	In vivo and in vitro	No	Yes	6 groups of 8 rats	Yes	Female rats are less affected than males by the three EDs chosen in this study. The modulation of the gene expression of kallikrein 4 and enamelin was higher in males than females.
2014	Jedeon et al. [32]	France	To assess the effects of BPA on ameloblasts and the potential involvement of the estrogen signaling pathway.	In vivo and in vitro	Yes	Yes	Male and female groups, each containing 16 control rats and 16 treated rats	Yes	Both BPA and estrogen stimulate the proliferation of ameloblasts via the estrogen receptor Erα (but not only). BPA impacts, preferentially, amelogenesis in male rats with a longer stage of secretion of ameloblasts and a shorter maturation stage.
2014	Jedeon et al. [33]	France	To assess the effect of the combination of several EDs (Genistein (G), Vinclozolin (V), and BPA) on tooth enamel.	In vivo and in vitro	Yes	No	6 groups of 8 rats	Yes	In vivo, the different combinations tested had less impact on enamel than BPA alone. In addition, the combination of G and/or V with BPA reduces the effects of BPA on enamel hypomineralization.
2013	Jedeon et al. [34]	France	To analyze the impact of BPA on amelogenesis.	In vivo and in vitro	Yes	No	16 treated rats and 16 control rats	Yes	The incisors of rats treated with BPA present, in 75% of cases, asymmetrical white spots at the enamel level with a phenotype similar to those of human MIH. BPA disrupts the normal removal of proteins from the enamel matrix (increased enamelin, decreased KLK4). There is a specific window of sensitivity.

BPA is the acronym for bisphenol A; ED means endocrine disruptor; KLK4 means kallikrein-related peptidase 4; G means Genistein; V means vinclozolin.

**Table 2:** Bias assessment with the use of Johanna Briggs Institute critical appraisal tools. The higher the score, the better the quality of the article.

Authors, Date	Bias Score
Winkler et al., 2024 [25]	62.5%
Liu et al., 2022 [26]	87.5%
Duman et al., 2022 [27]	87.5%
Ai Thu et al., 2017 (not evaluated, abstract only) [24]	-
Jedeon et al., 2016 (*J Bone & Min Res*) [28]	100%
Jedeon et al., 2016 (*Endocrinology*) [29]	100%
Houari et al., 2016 [30]	87.5%
Jedeon et al., 2016 (*Bull Group Int Rech Sto Od*) [31]	87.5%
Jedeon et al., 2014 (*Endocrinology*) [32]	100%
Jedeon et al., 2014 (*Connect Tissue Res*) [33]	100%
Jedeon et al., 2013 [34]	100%

## Data Availability

Data are available upon a reasonable request sent to the authors.

## Abbreviations

The following abbreviations are used in this manuscript:

BPA	Bisphenol A
BPS	Bisphenol S
ED	Endocrine disruptor
KLK4	Hypomineralized second primary molars
HSPM	Kallikrein-related peptidase 4
MIH	Molar incisor hypomineralization
REACH	Registration, Evaluation, Authorization and Restriction of Chemicals
SVHC	Substance of very high concern

## Appendix A

### Appendix A.1

**Table A1:** PubMed query completed on 26 May 2025. An “*” allows a search of all word endings to which it is affixed.

Query Terms	Number of Results
#1: ((((((((((((((((((((((((((((((((MIH [Title/Abstract])) OR (molar incisor [Title/Abstract])) OR (molar-incisor [Title/Abstract])) OR (molar incisor hypomineralisation [Title/Abstract])) OR (molar incisor hypomineralization [Title/Abstract])) OR (molar-incisor hypomineralization [Title/Abstract])) OR (molar incisor-hypomineralization [Title/Abstract])) OR (molar-incisor-hypomineralization [Title/Abstract])) OR (molar-incisor hypomineralization [Title/Abstract])) OR (molar-incisor-hypomineralization [Title/Abstract])) OR (molar incisor-hypomineralization [Title/Abstract])) OR (tooth hypomineralization [Title/Abstract])) OR (tooth hypomineralization [Title/Abstract])) OR (teeth hypomineralization [Title/Abstract])) OR (teeth hypomineralization [Title/Abstract])) OR (hypomineralization defect * [Title/Abstract])) OR (hypomineralization defect * [Title/Abstract])) OR (tooth enamel hypomineralization [Title/Abstract])) OR (teeth enamel hypomineralization [Title/Abstract])) OR (tooth enamel hypomineralization [Title/Abstract])) OR (teeth enamel hypomineralization [Title/Abstract])) OR (molar enamel hypomineralization [Title/Abstract])) OR (incisor enamel hypomineralization [Title/Abstract])) OR (molar incisor enamel hypomineralization [Title/Abstract])) OR (molar-incisor enamel hypomineralization [Title/Abstract])) OR (molar enamel hypomineralization [Title/Abstract])) OR (molar incisor enamel hypomineralization [Title/Abstract])) OR (molar incisor-enamel hypomineralization [Title/Abstract])) OR (incisor enamel hypomineralization [Title/Abstract])) OR (hspm [Title/Abstract])) OR (second primary molar hypomineralization [Title/Abstract])) OR (second primary molar hypomineralization [Title/Abstract])	1731
#2: (((((((((endocrine disrupt * [Title/Abstract]) OR (endocrine disrupting chemical * [Title/Abstract])) OR (EDC * [Title/Abstract])) OR (phenol * [Title/Abstract])) OR (Bisphenol a [Title/Abstract])) OR (BPA [Title/Abstract])) OR (Bisphenol-a [Title/Abstract])) OR (Bisphenol s [Title/Abstract])) OR (BPS [Title/Abstract])) OR (Bisphenol-s [Title/Abstract])	193,924
#3: (((review [Title]) OR review [Publication Type]) OR (meta-analysis [Publication Type])	3,936,087
#4: #1 AND #2	26
#5: #4 NOT #3	24

### Appendix A.2

**Table A2:** PRISMA checklist of the review.

Section and Topic	Item #	Checklist Item	Location Where Item Is Reported
**TITLE**			
Title	1	Identify the report as a systematic review.	Page 1
**ABSTRACT**			
Abstract	2	See the PRISMA 2020 for Abstracts checklist.	Page 1
**INTRODUCTION**			
Rationale	3	Describe the rationale for the review in the context of existing knowledge.	Pages 1 to 5
Objectives	4	Provide an explicit statement of the objective(s) or question(s) the review addresses.	Page 3
**METHODS**			
Eligibility criteria	5	Specify the inclusion and exclusion criteria for the review and how studies were grouped for the syntheses.	Page 3
Information sources	6	Specify all databases, registers, websites, organisations, reference lists and other sources searched or consulted to identify studies. Specify the date when each source was last searched or consulted.	Page 3
Search strategy	7	Present the full search strategies for all databases, registers and websites, including any filters and limits used.	Page 3
Selection process	8	Specify the methods used to decide whether a study met the inclusion criteria of the review, including how many reviewers screened each record and each report retrieved, whether they worked independently, and if applicable, details of automation tools used in the process.	Page 4
Data collection process	9	Specify the methods used to collect data from reports, including how many reviewers collected data from each report, whether they worked independently, any processes for obtaining or confirming data from study investigators, and if applicable, details of automation tools used in the process.	Page 4
Data items	10a	List and define all outcomes for which data were sought. Specify whether all results that were compatible with each outcome domain in each study were sought (e.g., for all measures, time points, analyses), and if not, the methods used to decide which results to collect.	Page 4
	10b	List and define all other variables for which data were sought (e.g., participant and intervention characteristics, funding sources). Describe any assumptions made about any missing or unclear information.	
Study risk of bias assessment	11	Specify the methods used to assess risk of bias in the included studies, including details of the tool(s) used, how many reviewers assessed each study and whether they worked independently, and if applicable, details of automation tools used in the process.	Page 4
Effect measures	12	Specify for each outcome the effect measure(s) (e.g., risk ratio, mean difference) used in the synthesis or presentation of results.	n/a
Synthesis methods	13a	Describe the processes used to decide which studies were eligible for each synthesis (e.g., tabulating the study intervention characteristics and comparing against the planned groups for each synthesis (item #5)).	Page 4
	13b	Describe any methods required to prepare the data for presentation or synthesis, such as handling of missing summary statistics, or data conversions.	Page 4
	13c	Describe any methods used to tabulate or visually display results of individual studies and syntheses.	
	13d	Describe any methods used to synthesize results and provide a rationale for the choice(s). If meta-analysis was performed, describe the model(s), method(s) to identify the presence and extent of statistical heterogeneity, and software package(s) used.	
	13e	Describe any methods used to explore possible causes of heterogeneity among study results (e.g., subgroup analysis, meta-regression).	
	13f	Describe any sensitivity analyses conducted to assess robustness of the synthesized results.	
Reporting bias assessment	14	Describe any methods used to assess risk of bias due to missing results in a synthesis (arising from reporting biases).	
Certainty assessment	15	Describe any methods used to assess certainty (or confidence) in the body of evidence for an outcome.	
**RESULTS**			
Study selection	16a	Describe the results of the search and selection process, from the number of records identified in the search to the number of studies included in the review, ideally using a flow diagram.	Page 4
	16b	Cite studies that might appear to meet the inclusion criteria, but which were excluded, and explain why they were excluded.	
Study characteristics	17	Cite each included study and present its characteristics.	Page 5
Risk of bias in studies	18	Present assessments of risk of bias for each included study.	Page 11
Results of individual studies	19	For all outcomes, present, for each study: (a) summary statistics for each group (where appropriate) and (b) an effect estimate and its precision (e.g., confidence/credible interval), ideally using structured tables or plots.	Pages 6 to 11
Results of syntheses	20a	For each synthesis, briefly summarise the characteristics and risk of bias among contributing studies.	
	20b	Present results of all statistical syntheses conducted. If meta-analysis was done, present for each the summary estimate and its precision (e.g., confidence/credible interval) and measures of statistical heterogeneity. If comparing groups, describe the direction of the effect.	
	20c	Present results of all investigations of possible causes of heterogeneity among study results.	
	20d	Present results of all sensitivity analyses conducted to assess the robustness of the synthesized results.	
Reporting biases	21	Present assessments of risk of bias due to missing results (arising from reporting biases) for each synthesis assessed.	
Certainty of evidence	22	Present assessments of certainty (or confidence) in the body of evidence for each outcome assessed.	
**DISCUSSION**			
Discussion	23a	Provide a general interpretation of the results in the context of other evidence.	Pages 11 to 13
	23b	Discuss any limitations of the evidence included in the review.	
	23c	Discuss any limitations of the review processes used.	
	23d	Discuss implications of the results for practice, policy, and future research.	
**OTHER INFORMATION**			
Registration and protocol	24a	Provide registration information for the review, including register name and registration number, or state that the review was not registered.	
	24b	Indicate where the review protocol can be accessed, or state that a protocol was not prepared.	
	24c	Describe and explain any amendments to information provided at registration or in the protocol.	
Support	25	Describe sources of financial or non-financial support for the review, and the role of the funders or sponsors in the review.	Page 13
Competing interests	26	Declare any competing interests of review authors.	Page 13
Availability of data, code and other materials	27	Report which of the following are publicly available and where they can be found: template data collection forms; data extracted from included studies; data used for all analyses; analytic code; any other materials used in the review.	Page 13
